# Caregiver Task Difficulties at the End of Life: A Latent Cluster Analysis

**DOI:** 10.1093/geroni/igab046.2850

**Published:** 2021-12-17

**Authors:** Kristin Litzelman, Irene Kizza, Ashley Berghoff

**Affiliations:** 1 University of Wisconsin-Madison, Madison, Wisconsin, United States; 2 University of Arkansas, Fayetteville, Arkansas, United States

## Abstract

Caregivers engage in myriad tasks from household help to complex medical care. However, little information is available on how caregivers experience individual tasks – particularly key end-of-life tasks such as managing breathing problems or patients' sadness and anxiety. The purpose of this study was therefore to assess task difficulty. Using data from the National Health and Aging Trends Survey and the National Survey on Caregivers (2015-2017), we assessed eleven caregiving tasks in 241 primary caregivers of care recipients in their last month of life. A latent cluster analysis revealed three key clusters: 1) pervasive difficulties, in which caregivers reported difficulty across most or all of the tasks; 2) minimal difficulties; and 3) emotional management difficulties, in which caregivers reported difficulty with managing sadness and anxiety and lower levels of difficulty on the other tasks. Weighted frequency analyses revealed that caregivers in the pervasive difficulties cluster were most likely to be filial caregivers (85% versus 63% of the full sample, p<0.05) or co-residing with the care recipient (49% versus 37% of the full sample, p<0.05). Caregivers identified as having pervasive difficulties were also more likely to report providing intensive care, more than 100 hours per week (54% versus 36% of the full sample, p<0.05). Care recipient condition was not associated with cluster membership. The findings highlight the need to consider caregiver coping at the task-level and have implications for understanding unmet needs. Future research will assess predictors of cluster membership and how task difficulties are associated with symptoms and well-being outcomes.

